# Whole genome sequencing data and *de novo* draft assemblies for 66 teleost species

**DOI:** 10.1038/sdata.2016.132

**Published:** 2017-01-17

**Authors:** Martin Malmstrøm, Michael Matschiner, Ole K. Tørresen, Kjetill S. Jakobsen, Sissel Jentoft

**Affiliations:** 1 Centre for Ecological and Evolutionary Synthesis (CEES), Department of Biosciences, University of Oslo, PO Box 1066, 0316 Oslo, Norway; 2 Centre for Coastal Research, Department of Natural Sciences, University of Agder, PO Box 422, 4604 Kristiansand, Norway

**Keywords:** Genome assembly algorithms, Comparative genomics, DNA sequencing

## Abstract

Teleost fishes comprise more than half of all vertebrate species, yet genomic data are only available for 0.2% of their diversity. Here, we present whole genome sequencing data for 66 new species of teleosts, vastly expanding the availability of genomic data for this important vertebrate group. We report on *de novo* assemblies based on low-coverage (9–39×) sequencing and present detailed methodology for all analyses. To facilitate further utilization of this data set, we present statistical analyses of the gene space completeness and verify the expected phylogenetic position of the sequenced genomes in a large mitogenomic context. We further present a nuclear marker set used for phylogenetic inference and evaluate each gene tree in relation to the species tree to test for homogeneity in the phylogenetic signal. Collectively, these analyses illustrate the robustness of this highly diverse data set and enable extensive reuse of the selected phylogenetic markers and the genomic data in general. This data set covers all major teleost lineages and provides unprecedented opportunities for comparative studies of teleosts.

## Background & Summary

Fueled by recent advances in comparative genomics, teleost fishes are becoming increasingly important research objects in several scientific disciplines, ranging from ecology, physiology and evolution to medicine, cancer research and aquaculture^1–7^. Genome information from non-model organisms is highly important in these comparative genomic analyses as they represent specific phenotypes that aid in disentangling the common parts of gene sets from those that have evolved as adaptations to specific ecosystems. In a quest to identify the evolutionary origin of the MHC II pathway loss first observed in the Atlantic cod (*Gadus morhua*)^8,9^, we applied a single sequencing library procedure to cost-efficiently produce draft assemblies for 66 teleost species, representing all major lineages within teleost fishes^10^. Since the alternative immune system, characterized by both the lack of MHC II and an expansion of MHC I, has so far only been identified in the Atlantic cod, we sampled the cod-like fishes of the order Gadiformes more densely than other groups, including 27 species of this order. Based on these genome sequence data, we were able to reconstruct the evolutionary history of the sampled lineages, to pinpoint the loss of the MHC II pathway to the common ancestor of all Gadiformes, and to identify several independent expansions in MHC I copy number within and outside the order Gadiformes. While these analyses and results are reported in a companion paper (Malmstrøm *et al.*^11^), we here present in greater detail the underlying data sets used for these analyses, including samples, sequencing reads (Data Citation 1), draft assemblies (Data Citation 2), and both mitochondrial and nuclear phylogenetic markers. By providing these data and the applied methodology in a coherent manner we aim to supply the scientific community with a highly diverse, reliable, and easy-to-use genomic resource for future comparative studies.

Our sequencing strategy was chosen on the basis of several pseudo-replicates of the budgerigar (*Melopsittacus undulatus*) genome^12^ (Data Citation 3), comprising different combinations of read lengths and coverages to determine the most cost-effective manner to produce genome data of sufficient quality for a reliable determination of gene presence or absence. These budgerigar data sets were furthermore assembled with two of the most used assemblers, the de Bruijn graph based SOAPdenovo^13^ and the Overlap-Layout-Consensus based Celera Assembler^14^ to investigate which assembly algorithm performed best on the various data replicates. On the basis of these *in silico* experiments, all species were sequenced on the Illumina HiSeq2000 platform, aiming for ~15× coverage. The sequenced reads were then quality controlled, error corrected and trimmed before performing assembly with Celera Assembler. The continuity of the assemblies was subsequently assessed through N50 statistics and the assembly quality was evaluated on the basis of gene space completeness of highly conserved genes. The assemblies were further used to identify mitochondrial genome sequences, which we used in combination with previously available sequences of related teleosts to verify the phylogenetic positions of sampled taxa (Data Citations 4 to 124). By recovering all taxa in their expected positions, clustering with conspecific or congeneric sequences where such were available, our phylogenetic analysis corroborates the correct identification of all sampled taxa and the absence of DNA contamination.

**Figure 1:**
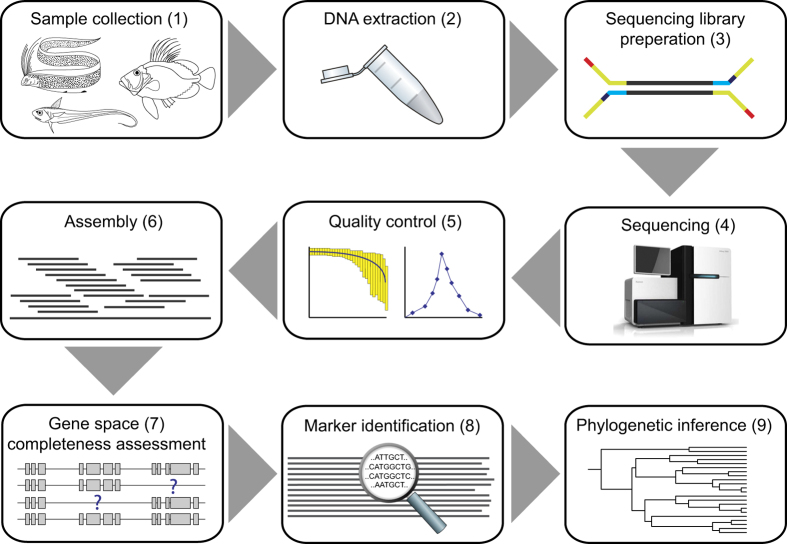
Flowchart illustrating the processes involved in creating and validating sequence data for 66 teleost species. (1) A full overview of species, sample supplier and tissue used for DNA extraction is provided in Table 1 (available online only). (2) The DNA extraction method is also found in Table 1 (available online only). (3) All sequencing libraries were created using the Illumina TruSeq Sample Prep v2 Low-Throughput Protocol. Adaptor indexes are provided in Table 2 (available online only). (4) Sequencing statistics and insert sizes for all species are also listed in Table 2 (available online only). (5) FastQC and SGA PreQC analyses were performed for all read sets prior to assembly. (6) Estimated genome sizes, coverages and assembly statistics for all species are presented in Table 3 (available online only), and accession links are provided in Table 4 (available online only). (7) CEGMA and BUSCO statistics are reported in Table 5 (available online only). (8) GenBank accession numbers and UTG IDs for all mitochondrial genomes used in phylogenetic analyses are provided in Tables 6 and 7 (available online only). (9) The maximum-likelihood phylogeny based on mitochondrial genomes is presented in Fig. 3.

Figure 1 illustrates the total workflow, and detailed information for each analysis step is further provided in the Methods section and in Tables 1–7 (available online only). The data sets presented here contain sequencing reads and assembled draft genomes for non-model species adapted to a wide variety of habitats, ranging from the deep sea and tropical coral reefs, to rivers and freshwater lakes. These data sets can be used individually or collectively, as resources for studies such as gene family evolution, adaptation to different habitats, phylogenetic inference of teleost orders, transposons and repeat content evolution as well as many other applications regarding gene and genome evolution in a comparative or model organism framework.

## Methods

### Sample acquisition and DNA extraction

The majority of samples were taken from validated species (mostly voucher specimens) and were provided by museums or university collections. Some samples were obtained from wild caught specimens, in collaboration with local fishermen. All samples were stored on either 96% ethanol or RNA-later (Ambion). The extraction of genomic DNA was carried out using either EZNA Tissue DNA Kit (Omega Bio-Tek), following the manufacturer’s instructions, or using the ‘High salt DNA extraction’ method as described by Phill Watts (https://www.liverpool.ac.uk/~kempsj/IsolationofDNA.pdf). Detailed information about all samples, including origin, voucher specimen ID and DNA extraction method is provided in Table 1 (available online only).

### Fragmentation and library preparation

Genomic DNA samples were diluted to 120 μl (50 ng μl^−1^) with Qiagen Elution Buffer (Qiagen) if necessary and fragmented to lengths of ~400 bp by sonication using a Covaris S220 (Life Technologies) with the following settings: 200 cycles for 90 s with ω-peak at 105. All sequencing libraries were constructed following the Illumina TruSeq Sample Prep v2 Low-Throughput Protocol.

### Sequencing and quality control

All sequencing was performed on an Illumina HiSeq 2000 platform with additional chemicals added to extend the number of cycles, yielding paired reads of 150 bp each. The read quality was then assessed using FastQC (http://www.bioinformatics.babraham.ac.uk/projects/fastqc/). Prior to assembly we used SGA PreQC^15^ to estimate coverage, per-base error rates, level of heterozygosity, repeat content and genome size in order to assess whether more sequencing would be needed. Some samples were then subjected to a second round of sequencing of the same library. Sequencing statistics are presented in Table 2 (available online only).

### Draft genome assembly

The methods used for genome assembly are also described in the Supplementary Note of Malmstrøm *et al.*^11^. We expand on these methods here, describing the different parameters and settings in greater detail in order to present a complete overview of our analyses.

All draft genomes were created using Celera Assembler, and the version used was downloaded from the CVS (Concurrent Version System, http://wgs-assembler.sourceforge.net/) repository on January 12th 2013. The program meryl, included in the Celera Assembler package was used to create a database of k-mers from the pairs of sequencing reads. Lower k-mer sizes might not resolve repetitive regions, while higher k-mer sizes might not overlap, leading to a loss of information required to correct the reads. Thus, an intermediate k-mer size of 22 was used for all assemblies. Meryl was run with the following options, where the sequences from the reads were concatenated into a file named ‘reads.fa’:

meryl -B -v -m 22 -memory 55000 -threads 16 -C -s reads.fa -o reads

In this command, –B specifies that a k-mer database should be created, and that this should be done using the verbose setting (-v). The –m option denotes the ‘merSize’, while –C specifies that canonical reads (both strands) should be used for creating the k-mer database.

The options –threads and –memory specify the computational resources that meryl can utilize and only influence run-time.

Most of the computational time used by Celera Assembler is required to identify overlap between reads. To reduce analysis time and generate longer input sequences, overlapping paired reads were merged with the software FLASH v1.2 (ref. 16), executed with the following command, where –d denotes the path to the output directory (with the prefix given with the –o option), –r is the read length, –f is the insert size, and –s is the standard deviation of the insert size:

flash input_1.fastq input_2.fastq -d. -r 150 -f 290 -s 50 –o output_prefix

Celera Assembler’s merTrim program (see Tørresen *et al.*^17^) was used to trim, error correct and remove adapters of all reads. The merTrim program estimates the coverage of the sequencing library by analysing the abundance of k-mers versus the number of k-mers at that abundance. By default, k-mers occurring at a frequency corresponding to at least one fourth of the coverage peak can be used to correct reads with k-mers that occur with a frequency of at most one third of the coverage peak. Reads were trimmed to the largest region containing k-mers with a frequency of more than one third of the coverage peak. The trimming of reads removes sequences not supported by other reads and reduces the possible fragmentation of the assembly. Adaptor sequences are not part of the genome and could lead to assembly fragmentation in the same way as repeated regions would. To remove adaptor sequences and other unsupported sequences from the read data, merTrim was executed with the following command:

merTrim -F reads.fastq -m 22 -mc meryl_db -mCillumina -t 16 -o out.fastq

In this command, –F specifies the reads, –m the k-mer size, –mc the database of trusted k-mers, and –mCillumina specifies that Illumina type adapters should be removed. The –t option defines the number of threads and thus only influences run time.

Following correction and trimming, the files in frg format were created with the following commands, as implemented in Celera Assembler:

fastqToCA -technology illumina -insertsize 500 50 -libraryname lib_name -mates read1_clean.fastq,read2_clean.fastq>paired_reads.frg

fastqToCA -technology illumina-long -insertsize 500 50 -libraryname lib_name -reads merged_reads.fastq>merged_reads.frg

The frg files contain information about the sequencing data, such as the expected insert size, location of the fastq files and the prefix for determining the species. Providing this information in the form of frg files is a prerequisite for Celera Assembler. Celera Assembler was then used to assemble the sequencing reads, with the following command specifying the prefix (–p) and the directory for the output (–d):

runCA -p prefix -d CA -s spec_file

The ‘spec_file’ contains a list of settings and run-options for Celera Assembler. Some of the settings and options are specific to the computing system used for the assembly (such as the number of parallel overlap processes, ‘ovlConcurrency’), but as mentioned above, k-mer size as specified with the option –m (‘merSize’) can have effects on the contiguity of the assembly. The option ‘doFragmentCorrection’ was set to 0 because the reads were corrected with merTrim. The content of this file was:

ovlConcurrency=4
ovlThreads=8
cnsConcurrency=32
merSize=22
merylMemory=50000
merylThreads=32
merThreshold=5000
doOBT=0
overlapper=ovl
ovlRefBlockSize=6000000
ovlHashBits=24
ovlHashBlockLength=800000000
doFragmentCorrection=0
unitigger=bogart
batMemory=55
batThreads=32
doExtendClearRanges=0
doToggle=0
paired_reads.frg
merged_reads.frg

The output of Celera Assembler consists of a set of three fasta files with increasing continuity that contain unitigs, contigs and scaffolds, respectively. Unitigs are either a unique DNA sequence found in a genome or a repeat, and unique unitigs are used as seeds to create contigs and scaffolds. In cases where Celera Assembler was not able to place a unitig confidently in the assembly, this unitig was not included in the contigs and scaffolds, but output separately. As a result of this, some additional sequence information is available in the assembled unitig fasta file compared to the assembled scaffolds. These additional sequences can include repeated sequences like transposable elements and tandem repeats, but also repeated gene fragments, conserved gene family domains, and other sequences that conflict with the biological assumptions of the assembler. As multiple copies of the mitochondrial genome are present in each cell, it is sequenced to a much higher coverage than the nuclear genome, and may therefore also be excluded from contigs due to false classification as a repetitive region. For these reasons, unitigs instead of contigs were used for both the identification of fragmented genes (see Malmstrøm *et al.*^11^) and for the mitochondrial phylogeny analysis described below. Assembly statistics for all draft genomes are provided in Table 3 (available online only).

### Code availability

The most crucial commands are implemented in the Methods section, while additional scripts (used in phylogenetic analyses) are available on the code repository on GitHub (https://github.com/uio-cees/teleost_genomes_data_descriptor).

## Data Records

All raw sequencing reads have been deposited in the European Nucleotide Archive (ENA) with study accession number PRJEB12469 (Data Citation 1). Table 4 (available online only) list the sample identifiers for each species. Each read file is available as a compressed file in fastq format (with extension fastq.gz). For some of the species, more than one read set is available as these were sequenced in two rounds, aiming to increase coverage. Two versions of all assembled genomes, unitigs (utg) and scaffolds (scf), are deposited in the Dryad repository under digital object identifier (DOI): doi:10.5061/dryad.326r8. (Data Citation 2). See Table 4 (available online only) for specific DOI for each species and assembly type.

## Technical Validation

**Figure 2:**
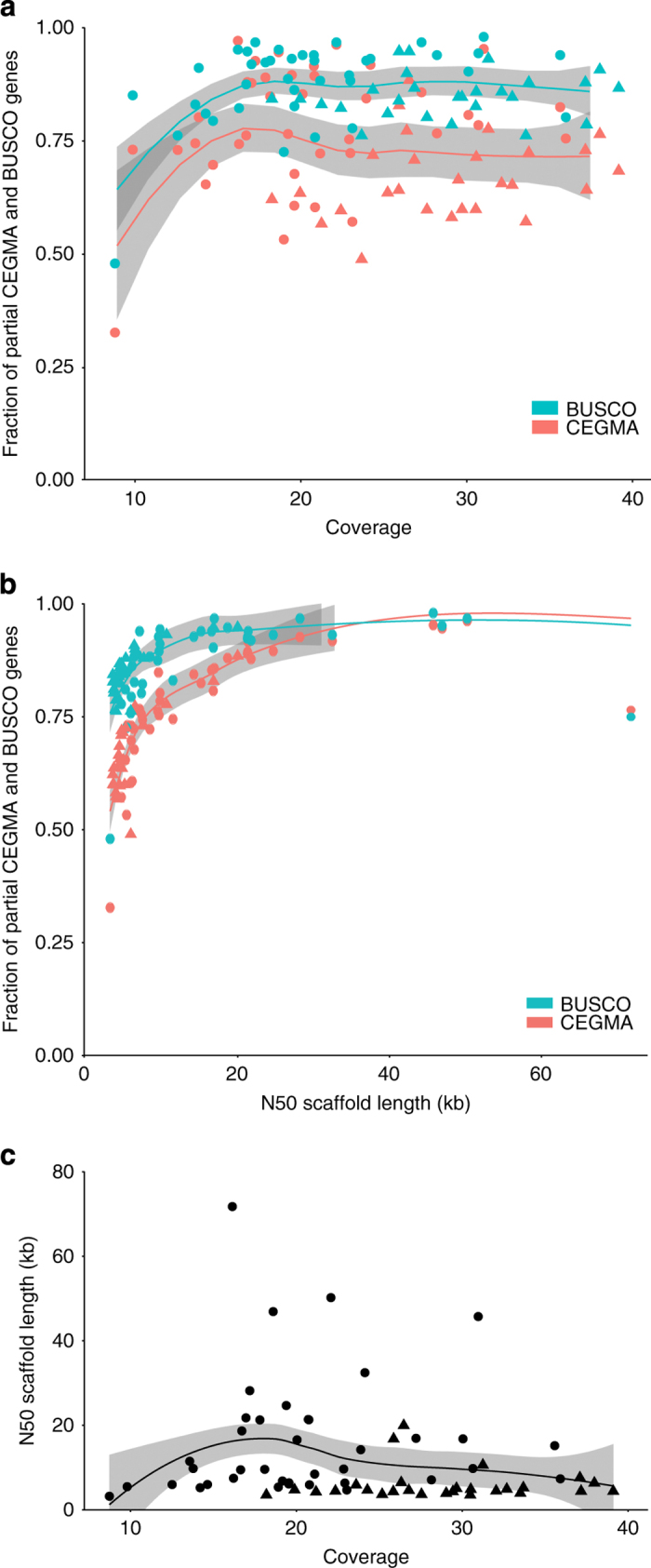
Correlation between gene space completeness, coverage, and N50 scaffold length for the 66 teleost genomes. (**a**) Scatterplot illustrating the correlation of gene space completeness (evaluated on the basis of BUSCO and CEGMA partially complete genes detected) and the read coverage (linear regression of BUSCO versus coverage (>15×): *R*^*2*^=0.038, *P*=0.07; CEGMA versus coverage (>15×): *R*^*2*^=0.002, *P*=0.30). (**b**) Scatterplot showing the correlation of BUSCO / CEGMA scores and N50 scaffold length (linear regression of BUSCO versus N50 scaffold length: *R*^*2*^=0.55, *P*<10^–12^ and CEGMA versus N50 scaffold length: *R*^*2*^=0.30, *P*<10^–5^) for all genome presented in the data set. (**c**) Scatterplot illustrating the correlation of coverage and N50 scaffold length (linear regression: *R*^*2*^=0.015, *P*=0.17). Species within the order Gadiformes are represented by triangles in all three plots. The lines shown are smooth LOESS curves, also referred to as local regressions, and the gray shaded areas represent 95% confidence interval in all three plots.

Both genome coverage and N50 lengths of contigs and scaffolds are considered important attributes for assessing a genome assembly. Assembly statistics for all species are reported in Table 2 (available online only). Another, and perhaps more crucial attribute, is the completeness of gene space, which is particularly important for the investigation of gene presence or absence. We used two different programs, CEGMA^18^ (Core Eukaryotic Genes Mapping Approach) v. 2.4.010312 and BUSCO^19^ (Benchmarking Universal Single-Copy Orthologs) v. 1.1b, to assess the gene-space completeness of our draft genome assemblies. CEGMA generates a list of ‘partial’ and ‘complete’ gene hits for the 248 most conserved genes, which were used as a validation of the assembly quality. BUSCO can be executed with several different reference data sets, optimized for different taxonomic groups. We used the ‘actinopterygii’ data set consisting of 3,698 highly conserved genes in acanthopterygian species (this specific data set is not publicly available yet—as of September 9th, 2016—but was provided by the developers of BUSCO upon request). BUSCO identifies and classifies these genes in the target genomes as either ‘Complete’, ‘Complete and duplicated’, ‘Fragmented’ or ‘Missing’. Table 5 (available online only) lists the CEGMA and BUSCO results for all assembled draft genomes, while Fig. 2a,b show the proportions of these conserved genes found (as partial hits) in relation to the read coverage and N50 scaffold length of all assemblies. In line with the results of our initial investigation of the budgerigar genome, we find no improvement in CEGMA or BUSCO gene set recovery when assembly coverage exceeds ~15× for the genomes included in this data set (linear regression of BUSCO versus coverage (>15×): *R*^*2*^=0.038, *P*=0.07; CEGMA versus coverage (>15×): *R*^*2*^=0.002, *P*=0.30) (Fig. 2a). When comparing the fractions of partial CEGMA and BUSCO genes recovered in each assembly with the N50 scaffold lengths of these assemblies, an initial steep increase is evident, clearly illustrating the sensitivity of these methods in relation to continuity (linear regression of BUSCO versus N50 scaffold length: *R*^*2*^=0.55, *P*<10^–12^; CEGMA versus N50 scaffold length: *R*^*2*^=0.30, *P*<10^–5^) (Fig. 2b). Finally, we find that the N50 scaffold length is largely uncorrelated with coverage (linear regression: *R*^*2*^=0.015, *P*=0.17), indicating that the specific sequencing strategy (insert size and read length) and the properties of the sequenced genomes (repeat content etc.) are more likely the limiting factors for N50 scaffold length (Fig. 2c). The observed lack of a correlation across all assemblies seems to be influenced by generally low N50 scaffold lengths for species of the order Gadiformes despite relatively high coverage for these genomes (mean coverage: 28×, mean N50 scaffold length: 6 kbp) compared to all other genomes (mean coverage: 20×, mean 50 scaffold length: 16 kbp). Thus, the species of the order Gadiformes appear more difficult to assemble which is likely explained by their high proportion of repetitive regions (see Tørresen *et al.*^17^). Collectively, these analyses illustrate that most of the variation in the recovery rate of the highly conserved genes is not due to low coverage, but rather reflects lineage-specific genomic features such as the amount and identity of repetitive elements that hamper the assembly of long continuous sequences.

### Phylogenetic analyses using mitochondrial genomes

To verify the correct identification of sampled species and the absence of contamination, we performed phylogenetic analyses of mitochondrial genomes extracted from all assemblies, in combination with previously available mitochondrial sequence data for sampled taxa and their close relatives. Mitochondrial genomes are particularly suitable for this comparison as the coverage of mitochondrial sequences is usually extremely high owing to the multiple copies of mitochondrial DNA (mtDNA) present in each mitochondrion and the large number of mitochondria per cell^20^. Furthermore, mitochondrial genomes are useful phylogenetic markers due to the very low frequency of recombination in animal mtDNA^21^ and the large number of mitochondrial genome sequences already available in GenBank^22^ (Data Citations 5 to 124).

We downloaded mitochondrial genome sequences for 120 species of which 14 species (*Lampris guttatus, Polymixia japonica, Percopsis transmontana, Zeus faber, Stylephorus chordatus, Lota lota, Gadus morhua, Monocentris japonicus, Rondeletia loricata, Beryx splendens, Antennarius striatus, Anabas testudineus, Helostoma temminkii, and Perca fluviatilis*) were also included in our set of 66 new teleost genome assemblies and an additional 8 species (*Osmerus mordax, Polymixia lowei, Bregmaceros nectabanus, Beryx decadactylus, Myripristis berndti, Lamprogrammus niger, Carapus bermudensis, and Thunnus thynnus*) were represented by a congener. GenBank accession numbers for the 120 downloaded genome sequences are given in Table 6 (available online only) (Data Citations 5 to 124). Protein-coding sequences for all mitochondrial genes except mt-ND6 (see Miya *et al.*^23^) were extracted from the 120 mitochondrial genomes, aligned with the software MAFFT^24^, v7.213 and translated to amino-acid sequences using AliView^25^ v.1.16.

To extract mitochondrial genomes from the 66 new unitig assemblies, we generated nucleotide BLAST databases for a subset of each assembly, consisting of all unitigs matched by at least 1,000 reads. This threshold was selected based on observed coverage distributions and the assumption that mitochondrial unitigs have particularly high coverage due to the relatively higher abundance of mitochondrial compared to nuclear DNA within each cell. The use of this threshold does not imply that all unitigs with higher coverage are mitochondrial, only that unitigs with lower coverage were ignored when mining for mitochondrial orthologs. For each mitochondrial gene, all 120 aligned amino-acid sequences were used as queries in searches with TBLASTN^26^ v.2.2.29 to identify unitigs with orthologous sequences in each of the 66 BLAST databases. For comparison, we also performed TBLASTN searches with the same queries against 10 additional BLAST databases generated for genome assemblies downloaded from ENSEMBL^27^ v.78 (*Danio rerio*, *Astyanax mexicanus*, *Gadus morhua*, *Gasterosteus aculeatus*, *Oreochromis niloticus*, *Oryzias latipes*, *Takifugu rubripes*, *Tetraodon nigroviridis* and *Xiphophorus maculatus*) and GenBank (*Salmo salar*; NCBI accession number AGKD00000000.3). For each of the 76 BLAST databases, the overall best TBLASTN hit for each mitochondrial gene was recorded and accepted as a homologous sequence if its e-value was below 1e^–15^. In cases where different unitigs matched different regions of the same gene (each with e-values below the threshold), these unitigs were jointly recorded as a single hit. Unitig identifiers for all hits are given in Table 7 (available online only). All hits were subsequently added to the untranslated mitochondrial gene alignments and realigned on the basis of amino-acid translations using TranslatorX^28^. Alignments were further analyzed with the software BMGE^29^ v.1.0 to determine unreliably aligned regions, and we excluded all codons that included sites with a gap rate above 0.2 or a smoothed entropy-like score (see Criscuolo & Gribaldo^29^) above 0.5. Finally, we concatenated the alignments of all mitochondrial genes, excluding two taxa (*Parasudis fraserbrunneri* and *Acanthochaenus luetkenii*) for which no homologs could be identified for eight or more genes. The final alignment used for phylogenetic inference included 9,303 bp.

Maximum-likelihood phylogenetic inference was performed with the software RAxML^30^ v.8.1.12, applying separate instances of the GTRCAT substitution model^31^ to three partitions corresponding to all first, second, and third codon positions. To assess the impact of potentially saturated third codon positions in the phylogenetic inference, we conducted two additional analyses in which these positions were either completely ignored or coded as ‘R’ and ‘Y’ so that only transversions would be counted as state changes. Phylogenetic node support was estimated through bootstrapping with an automatically determined number of bootstrap replicates (RAxML option ‘autoMRE’).

**Figure 3:**
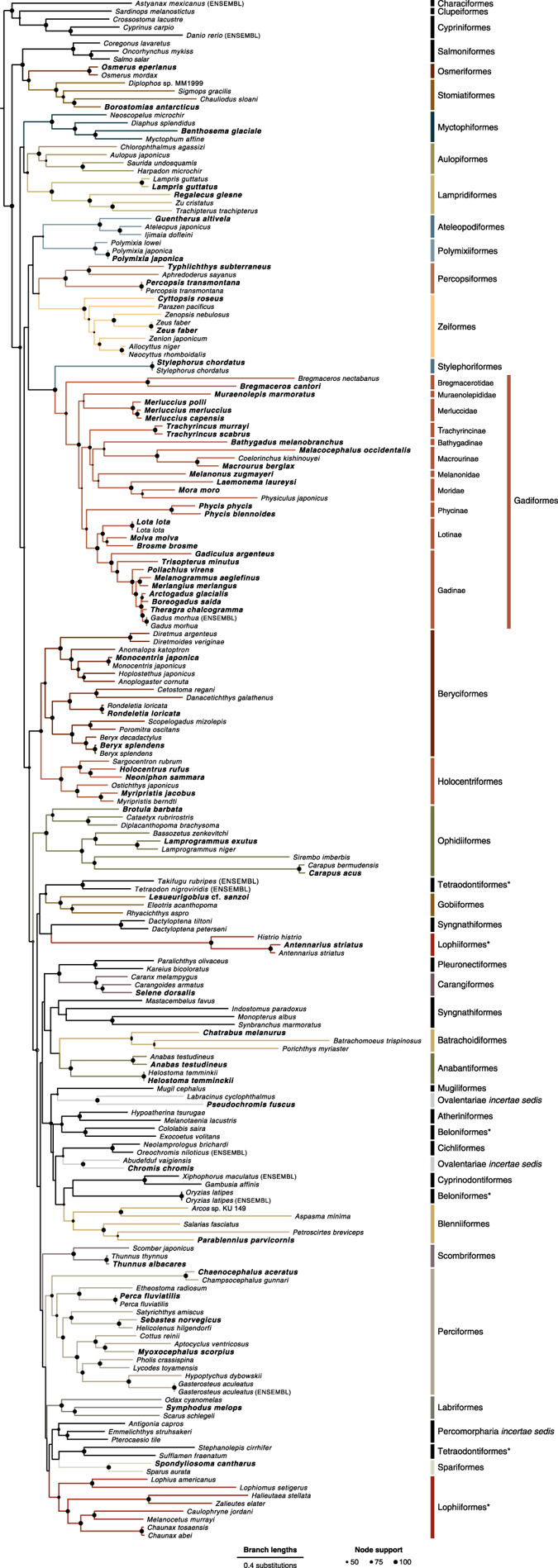
Maximum-likelihood phylogeny of teleost mitochondrial genome sequences. Sequences extracted from the new assemblies are marked in bold, all other mitochondrial genome sequences were previously available from the GenBank or ENSEMBL (where noted) databases. Black circles on nodes are sized proportional to bootstrap support, and the circle size corresponding to support values of 50, 75, and 100 are shown. Clade labels indicate taxonomic orders of all species as well as (with smaller font size) the (sub)family of gadiform species, following Betancur-R. *et al.*^10^ and Nelson^48^. Note that the orders Tetraodontiformes, Beloniformes, and Lophiiformes appear as non-monophyletic (marked with asterisks). For comparability, color code is identical to Fig. 1 in Malmstrøm *et al.*^11^. The tree file in Newick format has been deposited on Figshare under DOI: doi:10.6084/m9.figshare.4224234 (Data Citation 4).

Topologies of the three resulting maximum-likelihood phylogenies based on different usage of third codon positions were highly congruent, however, basal branches appeared to be best resolved in the analysis based on the alignment with three equally coded partitions. This maximum-likelihood phylogeny (Fig. 3) also received the highest mean bootstrap support (81.6, compared to 76.7 and 80.1 for the analyses in which third codon positions were ignored or coded as ‘R’ and ‘Y’, respectively). All taxa sampled for new genome assemblies had phylogenetic positions according to the expectations; for the 14 species for which we included both a GenBank sequence and a mitochondrial genome extracted from new assembly data, the two sequences clustered monophyletically in each case and were connected by short branches (see e.g., *Polymixia japonica*; Fig. 3). In other cases, mitochondrial genomes extracted from new assemblies clustered monophyletically with their congeneric counterparts downloaded from GenBank (see e.g., the mitochondrial genomes of *Osmerus eperlanus* and *Osmerus mordax*; Fig. 3).

It should be noted that basal phylogenetic nodes generally received relatively weak bootstrap support values, indicating that mitochondrial sequence data may not be sufficient to reliably resolve these ancient divergence events. Furthermore, three orders (Tetraodontiformes, Beloniformes, and Lophiiformes) appeared non-monophyletic, however, in all of these cases only weakly supported nodes separated two subgroups of the order. Thus, our results do not contradict the monophyly of these orders, which has been strongly supported in previous studies^10,32,33^. Most importantly, despite the not unexpected lower support values of basal nodes, our mitochondrial phylogeny corroborates the correct species identification and the absence of DNA contamination in the 66 new assemblies.

### Phylogenetic analyses using nuclear markers

To reliably reconstruct the evolutionary history of the 66 sequenced teleost species, we further extracted a set of carefully selected phylogenetic markers from the nuclear genomes. Based on a strict filtering procedure (see Malmstrøm *et al.*^11^), we selected one-to-one orthologs for 567 exons of 111 genes from the 66 draft assemblies and from 10 genome assemblies available in the ENSEMBL database (*Danio rerio*, *Astyanax mexicanus*, *Gasterosteus aculeatus*, *Oreochromis niloticus*, *Oryzias latipes*, *Takifugu rubripes*, *Tetraodon nigroviridis*, *Poecilia formosa* and *Xiphophorus maculatus*) or GenBank (*Salmo salar*). The 111 selected genes were characterized by clock-like evolution, homogeneity in GC content among species, and no or only weak signals of selection and were therefore particularly well suited for the reconstruction of time-calibrated phylogenies. The 111 genes were distributed across all chromosomes of the zebrafish genome and included between 3 and 14 exons that were used in our analyses. Per gene, we concatenated sequences of these exons into a single alignment, which then included between 300 and 1,888 (mean: 643.4) bp, between 47 and 777 (mean: 240.5) variable sites and between 33 and 490 (mean: 157.5) parsimony-informative sites. As orthologous sequences for the 111 genes could be detected in almost all assemblies, the resulting 111 alignments contained only between 1.4 and 11.9% (mean: 7.3%) missing data (Table 8 (available online only)).

**Figure 4:**
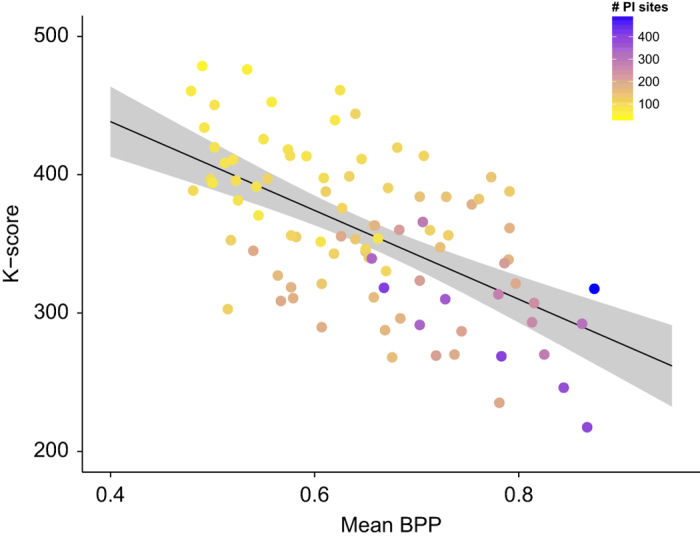
Distances between tree topologies compared to phylogenetic signal. Topological distances are measured by the *K*-score between the gene trees and the species trees, and phylogenetic signal of the gene trees is measured as mean Bayesian posterior probability (BPP). Dots are colored according to the number of parsimonious-informative (PI) sites. The black line represents the linear regression (*R*^*2*^=0.34, *P*<10^–15^).

These alignments were used for an extensive set of phylogenetic analyses to reconstruct the species tree as well as individual gene trees, using both maximum-likelihood and Bayesian inference. Detailed descriptions of these analyses and a discussion of the resulting species tree can be found in Malmstrøm *et al.*^11^ In addition, we here present analyses of gene tree discordance in relation to the 66 new assemblies, as a heterogeneous phylogenetic signal among gene trees could, among other causes (e.g. Fontaine *et al.*^34^; Gante *et al.*^35^), result from assembly issues such as contamination. To quantify gene tree discordance, we compared each gene tree to the species tree based on their *K*-scores^36^ and using the Shimodaira-Hasegawa (SH) test^37^ implemented in PAUP* v.4.0a150 (http://paup.csit.fsu.edu). All gene trees used in this comparison were maximum-clade-credibility (MCC) trees inferred with the software BEAST^38^ v.2.2.0 for each of the 111 alignments. Similarly, we considered the MCC tree inferred with BEAST for a single concatenated alignment of all genes as the species tree (Fig. 1 in Malmstrøm *et al.*^11^) used in this comparisons. According to results of the SH test, all but four gene trees were significantly different (*P*<0.05) from the species tree (Table 8 (available online only)). *K*-scores were calculated for 91 of the 111 gene trees, but could not be calculated for the remaining 20 gene trees due to negative branch lengths. The resulting *K*-scores ranged from 217.5 to 478.6, indicating considerable gene tree discordance in agreement with the results of the SH test (even though individual *K*-scores and SH test *P*-values did not correlate; *P*=0.64). However, such tree discordance does not necessarily indicate assembly issues but can arise from multiple factors including incomplete lineage sorting^39^ or a lack of phylogenetic signal^40^. While high levels of incomplete lineage sorting have been shown to affect phylogenomic inference of rapidly radiating lineages like Neoavian birds^41^ or cichlid fishes^42,43^, its effect is expected to be limited in the analysis of ancient clades with long internode distances^44^ such as the teleost species tree inferred from our set of 111 nuclear markers^11^. We investigated the presence of incomplete lineage sorting in this species tree in Malmstrøm *et al.*^11^ by testing for a correlation of indel hemiplasy and branch length^45^. However, since no such correlation could be detected in our data set, we concluded that incomplete lineage sorting was weak or absent in the teleost species tree reported in Malmstrøm *et al.*^11^. In addition, we now tested whether instead of incomplete lineage sorting, a lack of phylogenetic signal in individual marker alignments could explain the observed gene tree discordance. To this end, we calculated the mean Bayesian posterior probability (BPP) of each gene tree as a measure of its phylogenetic signal and compared it to the *K*-score between this gene tree and the species tree. We find a highly significant negative correlation between the two measures (linear regression: *R*^*2*^=0.34, *P*<10^–15^), which is illustrated in Fig. 4. Furthermore, we also detected a highly significant correlation between the number of parsimony-informative sites per marker and the respective *K*-score (linear regression: *R*^2^=0.49, *P*<10^–13^) (Fig. 4). These tests show that low phylogenetic signal in individual marker alignments, rather than contamination in the assemblies, is responsible for the observed gene tree discordance. This lack of signal in individual alignments, however, is not exclusive to our phylogenomic data set, but is a feature that is commonly observed in nuclear markers^40,44^. As demonstrated by Malmstrøm *et al.*^11^ as well as other phylogenomic studies^45–47^ the combination of such stringently filtered exonic markers nevertheless allows an extremely reliable inference of ancient species trees that could not be achieved with faster-evolving sequence such as mitochondrial genomes, intronic regions, or genes under selection. We therefore recommend the reuse of the marker set presented here as a highly suitable resource for future analyses of the teleost species tree with extended taxon sets.

## Usage Notes

Sequencing reads from all species can be downloaded from the European Nucleotide Archive (ENA), under the sample identifiers ERS1199874—ERS1199939. Unitig and scaffold level assemblies are available for download from the Dryad repository with individual assemblies found under DOI: doi:10.5061/dryad.326r8/1.—dryad.326r8/132. See Table 7 (available online only) for individual identifiers for both the raw sequencing read sets and the two assembly versions. The mitochondrial phylogeny (Fig. 3) can be downloaded as a tree file in Newick format from Figshare under DOI: doi:10.6084/m9.figshare.4224234 (Data Citation 4).

## Additional information

**How to cite**: Malmstrøm, M. *et al.* Whole genome sequencing data and *de novo* draft assemblies for 66 teleost species. *Sci. Data* 4:160132 doi: doi:10.1038/sdata.2016.132 (2017).

**Publisher’s note**: Springer Nature remains neutral with regard to jurisdictional claims in published maps and institutional affiliations.

## Supplementary Material



## Figures and Tables

**Table 1:** Sample information for all species in the reported data set

**Order**	**Species**	**Tissue**	**Sample ID**	**Voucher ID**
Osmeriformes	*Osmerus eperlanus*^*1*^	Fin*	Osep_1_#2	NA
Stomiatiformes	*Borostomias antarcticus*^*2*^	Muscle†	JYP 598	ZMUC 8046
Aulopiformes	*Parasudis fraserbrunneri*^*3*^	Muscle*	A430	CFM 117870
Ateleopodiformes	*Guentherus altivela*^*3*^	Muscle*	B375	NA
Myctophiformes	*Benthosema glaciale*^*2*^	Muscle†	JYP 403	ZMUC 8477
Polymyxiformes	*Polymixia japonica*^*4*^	Muscle*	NSMT-P 79586.1	NSMTNAP 79586
Percopsiformes	*Percopsis transmontana*^*5*^	Muscle*	KU:KUIT:1890	KU:KUI:29775
Percopsiformes	*Typhlichthys subterraneus*^*5*^	Muscle*	KU:KUIT:8754	UAIC 14148.01
Zeiformes	*Zeus faber*^*3*^	Muscle*	B11	ZSCM 32795
Zeiformes	*Cyttopsis roseus*^*3*^	Muscle†	B361	ZSCM 32479
Stylephoriformes	*Stylephorus chordatus*^*5*^	Muscle*	KU:KUIT:8138	MCZ 165920
Gadiformes	*Bregmaceros cantori*^*5*^	Muscle†	KU:KUIT:5133	KU:KUI:30244
Gadiformes	*Merluccius polli*^*3*^	Muscle†	B116	ZSCM 40336
Gadiformes	*Merluccius merluccius*^*6*^	Thymus*	Meme(Ly)_IOF_1_#2	NA
Gadiformes	*Merluccius capensis*^*3*^	Muscle†	B16	ZSCM 32773
Gadiformes	*Melanonus zugmayeri*^*3*^	Muscle*	B304	ZSCM 32519
Gadiformes	*Muraenolepis marmoratus*^*3*^	Muscle*	#95	NA
Gadiformes	*Trachyrincus scabrus*^*3*^	Muscle*	A35	CFM 117888
Gadiformes	*Trachyrincus murrayi*^*7*^	Fin*	A9-2012-420-171-1	NA
Gadiformes	*Mora moro*^*8*^	Muscle*	Momo(Dy)_Sula_4_#1	NA
Gadiformes	*Laemonema laureysi*^*3*^	Muscle†	B43	ZSCM 32710
Gadiformes	*Bathygadus melanobranchus*^*3*^	Muscle*	B365	ZSCM 40344
Gadiformes	*Macrourus berglax*^*6*^	Muscle*	Mabe_1_#1	NA
Gadiformes	*Malacocephalus occidentalis*^*3*^	Muscle*	A25	CFM 117884
Gadiformes	*Phycis blennoides*^*7*^	Fin*	A9-2012-418-76-1	NA
Gadiformes	*Phycis phycis*^*3*^	Muscle†	Phph_X5	NA
Gadiformes	*Lota lota*^*3*^	Muscle*	Lolo_X10	NA
Gadiformes	*Molva molva*^*6*^	Thymus*	Momo(Br)_IOF_1_#2	NA
Gadiformes	*Brosme brosme*^*6*^	Spleen*	Brbr_LO_1_#2	NA
Gadiformes	*Trisopterus minutus*^*6*^	Spleen†	Trmi_IOF_1_#1	NA
Gadiformes	*Gadiculus argenteus*^*6*^	Spleen†	Gaar_IOF_1_#2	NA
Gadiformes	*Pollachius virens*^*6*^	Spleen†	Povi_LO_1_#1	NA
Gadiformes	*Melanogrammus aeglefinus*^*6*^	Spleen†	Meae_LO_1_#2	NA
Gadiformes	*Merlangius merlangus*^*6*^	Thymus*	Meme(Hy)_OOF_1_#2	NA
Gadiformes	*Arctogadus glacialis*^*9*^	Fin†	0A-08-045_#3	NA
Gadiformes	*Boreogadus saida*^*7*^	Fin*	B3-2012-189-6_#1	NA
Gadiformes	*Theragra chalcogramma*^*10*^	Fin†	HS-08.010_#1	NA
Gadiformes	*Gadus morhua*^*6*^	Blood‡	NEAC_001	NA
Lampriformes	*Regalecus glesne*^*3*^	Muscle*	Regl_X3	NA
Lampriformes	*Lampris guttatus*^*3*^	Muscle*	Lagu_X8	NA
Beryciformes	*Monocentris japonica*^*4*^	Muscle*	NSMT-P 75883.1	NSMTNAP 75883
Holocentriformes	*Myripristis jacobus*^*3*^	Blood*	KV124	NA
Holocentriformes	*Holocentrus rufus*^*3*^	Blood†	X2	NA
Holocentriformes	*Neoniphon sammara*^*5*^	Muscle*	KU:KUIT:6925	SAIAB 77852
Beryciformes	*Beryx splendens*^*3*^	Muscle†	A252	NA
Beryciformes	*Rondeletia loricata*^*5*^	Muscle*	KU:KUIT:8426	MCZ 167869
Beryciformes	*Acanthochaenus luetkenii*^*5*^	Muscle†	KU:KUIT:8430	MCZ 167873
Ophidiiformes	*Brotula barbata*^*3*^	Muscle*	B392	ZSCM 32626
Ophidiiformes	*Lamprogrammus exutus*^*3*^	Muscle†	A69	CFM 118100
Ophidiiformes	*Carapus acus*^*3*^	Muscle†	B358	ZSCM 32503
Batrachoidiformes	*Chatrabus melanurus*^*3*^	Muscle†	B25	ZSCM 32594
Scombriformes	*Thunnus albacares*^*3*^	Muscle†	Sp569	NA
Gobiiformes	*Lesueurigobius* cf. *sanzi*^*3*^	Muscle*	B265	NA
Perciformes	*Perca fluviatilis*^*3*^	Muscle†	Pefl_NEG_1_#1	NA
Perciformes	*Myoxocephalus scorpius*^*3*^	Muscle*	Seescorpion_1	NA
Perciformes	*Sebastes norvegicus*^*6*^	Spleen†	Seno_LO_1_#1	NA
Perciformes	*Chaenocephalus aceratus*^*9*^	Muscle†	ANT-XXVII/3,#299	NA
Labriformes	*Symphodus melops*^*6*^	Muscle*	Kg47	NA
Spariformes	*Spondyliosoma cantharus*^*3*^	Muscle*	Sp28	NA
Lophiiformes	*Antennarius striatus*^*3*^	Muscle†	B133	ZSCM 32591
Carangiformes	*Selene dorsalis*^*3*^	Muscle†	B97	NA
Anabantiformes	*Helostoma temminckii*^*11*^	Muscle*	LR10256	NA
Anabantiformes	*Anabas testudineus*^*11*^	Muscle†	LR11643	NA
Blenniiformes	*Parablennius parvicornis*^*11*^	Muscle*	LR00424	NA
Ovalentariae* incertae sedis*	*Chromis chromis*^*3*^	Muscle*	Sp51	NA
Ovalentariae* incertae sedis*	*Pseudochromis fuscus*^*12*^	Muscle*	F5-C4_1	NA
ZSCM numbers are vouchers from Zoological State Collection Munich				
CFM numbers are vouchers from Chicago Field Museum collection				
ZMUC number refers to voucher from Zoological Museum University of Copenhagen collection				
KUI number refer to voucher from University of Kansas Biodiversity Institute Icthyology collection				
UAIC number refers to voucher from University of Alabama Ichtyology collection				
SAIAB number refers to voucher from South African Institute for Aquatic Biodiversity collection				
MCZ number refer to voucher from Museum of Comparative Zoology, Harvard University collection				
NSMT-P number refer to voucher from National Museum of Nature and Science, Tsukuba, Japan				
^1^Kjartan Østbye (University of Oslo, Norway), ^2^Jan Yde Poulsen (Greenland Institute of Natural Resources, Greenland), ^3^Reinhold Hanel (Thünen-Institute of Fisheries Ecology, Germany), ^4^Masaki Miya (Natural History Museum & Institute in Chiba, Japan), ^5^Andrew Bentley (University of Kansas Biodiversity Institute, USA), ^6^Martin Malmstrøm (University of Oslo, Norway), ^7^Christophe Pampoulie (Marine Research Institute of Iceland, Iceland), ^8^Irvin Kilde (NorwegianUniversity of Science and Technology in Trondheim, Norway), ^9^Walter Salzburger (University of Basel, Switzerland), ^10^Ian Bradbury (Memorial university, Canada), ^11^Lukas Rüber (Natural History Museum in Bern, Switzerland), ^12^Fabio Cortesi (University of Queensland, Australia)				

*Isolated with EZNA spin columns

^†^Isolated with high salt method

^‡^Isolated with blood plug protocol (See Star *et al.*)^8^

**Table 2:** Sequencing information for all species in the reported data set (Data Citation 1)

**Species**	N reads*	**N bases**	**Bases used**†	**Insert size**‡	**Adaptor index**
*Osmerus eperlanus*	84.7	12,709	74%	366	AD023
*Borostomias antarcticus*	131.9	19,789	83%	439	AD002
*Parasudis fraserbrunneri*	149.6	22,436	82%	366	AD022
*Guentherus altivela*	101.7	15,256	97%	380	AD020
*Benthosema glaciale*	194.4	29,154	87%	453	AD002
*Polymixia japonica*	90.2	13,525	85%	367	AD016
*Percopsis transmontana*	147.8	22,168	82%	350	AD001
*Typhlichthys subterraneus*	142.7	21,398	81%	336	AD003
*Zeus faber*	151.5	22,722	74%	335	AD008
*Cyttopsis roseus*	161.0	24,156	75%	340	AD013
*Stylephorus chordatus*	171.7	25,752	87%	428	AD014
*Bregmaceros cantori*	310.3	46,537	84%	343	AD013
*Merluccius polli*	112.9	16,931	80%	353	AD015
*Merluccius merluccius*	146.5	21,968	82%	359	AD013
*Merluccius capensis*	107.0	16,047	81%	353	AD014
*Melanonus zugmayeri*	179.2	26,884	81%	351	AD016
*Muraenolepis marmoratus*	127.3	19,101	80%	345	AD009
*Trachyrincus scabrus*	179.5	26,928	82%	358	AD008
*Trachyrincus murrayi*	66.8	20,050	89%	519	AD014
*Mora moro*	153.0	22,945	85%	345	AD014
*Laemonema laureysi*	105.7	15,862	80%	348	AD015
*Bathygadus melanobranchus*	122.4	18,362	83%	330	AD008
*Macrourus berglax*	119.7	17,948	82%	356	AD001
*Malacocephalus occidentalis*	140.8	21,117	78%	352	AD003
*Phycis blennoides*	80.6	24,191	87%	505	AD015
*Phycis phycis*	130.9	19,631	76%	328	AD005
*Lota lota*	125.1	18,758	83%	341	AD007
*Molva molva*	152.3	22,852	79%	329	AD006
*Brosme brosme*	122.5	18,373	80%	342	AD012
*Trisopterus minutus*	151.6	22,737	76%	325	AD005
*Gadiculus argenteus*	138.1	20,709	79%	320	AD004
*Pollachius virens*	112.4	16,854	79%	328	AD006
*Melanogrammus aeglefinus*	110.9	16,639	82%	324	AD007
*Merlangius merlangus*	165.2	24,787	85%	339	AD012
*Arctogadus glacialis*	165.2	22,006	81%	323	AD002
*Boreogadus saida*	155.1	25,243	77%	316	AD004
*Theragra chalcogramma*	155.1	23,268	84%	322	AD002
*Gadus morhua*	62.8	18,847	92%	531	AD019
*Regalecus glesne*	183.9	27,578	83%	344	AD027
*Lampris guttatus*	162.7	24,407	82%	353	AD010
*Monocentris japonica*	90.5	13,580	87%	468	AD012
*Myripristis jacobus*	64.3	19,274	84%	353	AD001
*Holocentrus rufus*	61.5	18,463	83%	354	AD003
*Neoniphon sammara*	90.9	13,631	87%	474	AD005
*Beryx splendens*	90.1	13,509	97%	465	AD005
*Rondeletia loricata*	127.8	19,167	89%	467	AD004
*Acanthochaenus luetkenii*	129.4	19,411	90%	430	AD014
*Brotula barbata*	131.8	19,769	81%	352	AD015
*Lamprogrammus exutus*	63.3	9,500	93%	348	AD014
*Carapus acus*	102.7	15,410	79%	349	AD016
*Chatrabus melanurus*	333.0	49,954	82%	353	AD020
*Thunnus albacares*	120.5	18,075	86%	461	AD015
*Lesueurigobius* cf*. sanzi*	66.2	19,846	92%	529	AD018
*Perca fluviatilis*	96.9	14,532	78%	364	AD025
*Myoxocephalus scorpius*	105.7	15,847	80%	423	AD019
*Sebastes norvegicus*	131.0	19,653	80%	349	AD027
*Chaenocephalus aceratus*	301.2	45,178	83%	355	AD022
*Symphodus melops*	86.4	12,694	86%	440	AD019
*Spondyliosoma cantharus*	97.6	14,642	90%	443	AD014
*Antennarius striatus*	71.6	10,742	71%	363	AD022
*Selene dorsalis*	107.9	16,179	86%	458	AD016
*Helostoma temminckii*	85.3	12,795	87%	464	AD016
*Anabas testudineus*	96.8	14,520	88%	459	AD016
*Parablennius parvicornis*	141.9	21,288	88%	459	AD019
*Chromis chromis*	166.1	24,917	87%	341	AD018
*Pseudochromis fuscus*	54.7	16,404	87%	418	AD018

*In millions

^†^Percentage of total bases used in Celera assembly

^‡^After merging with FLASH (bp)

**Table 3:** Assembly statistics for all species in the reported data set (Data Citation 2).

**Species**	Genome size* **(Mb)**	**Coverage**†	**N50 contigs length (bp)**	**N50 scaffold length (bp)**	**Total span of scaffolds (Mb)**	**Recovered**‡
*Osmerus eperlanus*	489	19.16	4,524	6,798	342	70%
*Borostomias antarcticus*	865	18.91	3,928	5,352	429	50%
*Parasudis fraserbrunneri*	935	19.55	4,177	6,366	706	76%
*Guentherus altivela*	1,701	8.73	2,928	3,199	538	32%
*Benthosema glaciale*	1,304	19.54	4,393	6,091	674	52%
*Polymixia japonica*	635	18.09	5,803	9,534	553	87%
*Percopsis transmontana*	509	35.57	8,161	15,134	457	90%
*Typhlichthys subterraneus*	759	22.85	7,314	9,640	555	73%
*Zeus faber*	732	22.92	4,642	6,313	609	83%
*Cyttopsis roseus*	640	28.14	4,843	7,060	545	85%
*Stylephorus chordatus*	971	23.04	3,373	4,661	487	50%
*Bregmaceros cantori*	1,650	23.60	4,452	5,909	1,142	69%
*Merluccius polli*	609	22.35	3,471	4,468	400	66%
*Merluccius merluccius*	611	29.65	3,670	5,094	400	66%
*Merluccius capensis*	653	19.89	3,792	4,760	413	63%
*Melanonus zugmayeri*	589	37.09	4,562	7,599	432	73%
*Muraenolepis marmoratus*	840	18.19	3,126	3,549	415	49%
*Trachyrincus scabrus*	579	37.96	3,900	6,346	369	64%
*Trachyrincus murrayi*	678	26.47	6,231	19,931	450	66%
*Mora moro*	499	39.10	3,267	4,412	344	69%
*Laemonema laureysi*	524	24.28	3,431	4,696	305	58%
*Bathygadus melanobranchus*	577	26.32	4,956	6,466	430	75%
*Macrourus berglax*	693	21.18	3,353	4,278	399	58%
*Malacocephalus occidentalis*	504	32.68	3,697	4,907	349	69%
*Phycis blennoides*	674	31.24	4,532	10,570	414	61%
*Phycis phycis*	468	32.04	3,458	4,486	345	74%
*Lota lota*	512	30.51	3,803	4,876	397	78%
*Molva molva*	539	33.68	4,136	5,251	437	81%
*Brosme brosme*	551	26.78	3,682	4,636	412	75%
*Trisopterus minutus*	517	33.50	3,248	3,962	334	65%
*Gadiculus argenteus*	567	29.03	3,379	3,942	396	70%
*Pollachius virens*	513	25.84	3,457	4,331	394	77%
*Melanogrammus aeglefinus*	543	25.17	3,215	3,690	374	69%
*Merlangius merlangus*	566	37.16	3,538	4,430	423	75%
*Arctogadus glacialis*	646	27.54	3,282	3,696	429	66%
*Boreogadus saida*	641	30.48	3,221	3,566	412	64%
*Theragra chalcogramma*	661	29.43	3,603	4,323	448	68%
*Gadus morhua*	674	25.86	5,765	16,731	492	73%
*Regalecus glesne*	750	30.64	6,781	9,753	654	87%
*Lampris guttatus*	1,405	14.20	4,051	5,212	847	60%
*Monocentris japonica*	706	16.71	8,046	18,610	554	79%
*Myripristis jacobus*	778	20.75	9,816	21,260	719	92%
*Holocentrus rufus*	735	20.72	9,243	21,323	648	88%
*Neoniphon sammara*	696	16.96	8,761	21,687	657	94%
*Beryx splendens*	897	14.64	4,286	5,972	532	59%
*Rondeletia loricata*	1,049	16.21	5,112	7,444	567	54%
*Acanthochaenus luetkenii*	825	21.10	5,636	8,398	544	66%
*Brotula barbata*	519	30.96	17,578	45,713	484	93%
*Lamprogrammus exutus*	901	9.80	4,213	5,459	492	55%
*Carapus acus*	448	27.21	9,554	16,897	387	86%
*Chatrabus melanurus*	1,965	20.79	4,581	5,906	1,126	57%
*Thunnus albacares*	836	18.60	16,808	46,871	726	87%
*Lesueurigobius* cf.* sanzi*	1,349	13.57	6,729	11,439	808	60%
*Perca fluviatilis*	903	12.51	4,140	5,951	629	70%
*Myoxocephalus scorpius*	759	16.64	5,716	9,443	518	68%
*Sebastes norvegicus*	782	20.04	9,467	16,530	716	92%
*Chaenocephalus aceratus*	1,050	35.91	5,460	7,309	623	59%
*Symphodus melops*	628	17.80	9,362	21,217	533	85%
*Spondyliosoma cantharus*	767	17.19	11,633	28,109	679	89%
*Antennarius striatus*	552	13.78	6,086	9,743	441	80%
*Selene dorsalis*	576	24.13	11,209	32,351	527	92%
*Helostoma temminckii*	686	16.14	17,055	71,662	599	87%
*Anabas testudineus*	576	22.08	18,817	50,098	524	91%
*Parablennius parvicornis*	623	30.04	7,343	16,734	598	96%
*Chromis chromis*	907	23.88	8,509	14,185	832	92%
*Pseudochromis fuscus*	740	19.39	12,029	24,629	656	89%

*Estimated by Celera Assembler

^†^Based on Celera Assembler genome estimation

^‡^Span of scaffolds divided by estimated genome size

**Table 4:** Individual identifiers for samples (read sets) in ENA and genome assemblies in the Dryad repository (Data Citation 2)

**Species**	**ENA sample accession**	**DOI for scaffold assembly**	**Size (Mb)**	**DOI for unitig assembly**	**Size (Mb)**
*Osmerus eperlanus*	SAMEA4028764	doi:10.5061/dryad.326r8/81.	104.5	doi:10.5061/dryad.326r8/82.	207.1
*Borostomias antarcticus*	SAMEA4028765	doi:10.5061/dryad.326r8/98.	128.6	doi:10.5061/dryad.326r8/90.	460.2
*Parasudis fraserbrunneri*	SAMEA4028766	doi:10.5061/dryad.326r8/71.	213.6	doi:10.5061/dryad.326r8/72.	463.8
*Guentherus altivela*	SAMEA4028767	doi:10.5061/dryad.326r8/77.	165.8	doi:10.5061/dryad.326r8/78.	858.8
*Benthosema glaciale*	SAMEA4028768	doi:10.5061/dryad.326r8/91.	204.8	doi:10.5061/dryad.326r8/92.	684.3
*Polymixia japonica*	SAMEA4028769	doi:10.5061/dryad.326r8/47.	167.5	doi:10.5061/dryad.326r8/48.	282.7
*Percopsis transmontana*	SAMEA4028770	doi:10.5061/dryad.326r8/49.	140.0	doi:10.5061/dryad.326r8/50.	194.1
*Typhlichthys subterraneus*	SAMEA4028771	doi:10.5061/dryad.326r8/51.	169.5	doi:10.5061/dryad.326r8/52.	286.6
*Zeus faber*	SAMEA4028772	doi:10.5061/dryad.326r8/53.	186.1	doi:10.5061/dryad.326r8/54.	362.3
*Cyttopsis roseus*	SAMEA4028773	doi:10.5061/dryad.326r8/55.	166.8	doi:10.5061/dryad.326r8/56.	294.8
*Stylephorus chordatus*	SAMEA4028774	doi:10.5061/dryad.326r8/103.	147.4	doi:10.5061/dryad.326r8/104.	455.1
*Bregmaceros cantori*	SAMEA4028775	doi:10.5061/dryad.326r8/41.	341.4	doi:10.5061/dryad.326r8/42.	879.0
*Merluccius polli*	SAMEA4028776	doi:10.5061/dryad.326r8/29.	121.2	doi:10.5061/dryad.326r8/30.	295.7
*Merluccius merluccius*	SAMEA4028777	doi:10.5061/dryad.326r8/25.	121.2	doi:10.5061/dryad.326r8/26.	328.6
*Merluccius capensis*	SAMEA4028778	doi:10.5061/dryad.326r8/27.	125.1	doi:10.5061/dryad.326r8/28.	351.8
*Melanonus zugmayeri*	SAMEA4028779	doi:10.5061/dryad.326r8/31.	130.3	doi:10.5061/dryad.326r8/32.	331.4
*Muraenolepis marmoratus*	SAMEA4028780	doi:10.5061/dryad.326r8/39.	123.4	doi:10.5061/dryad.326r8/40.	398.6
*Trachyrincus scabrus*	SAMEA4028781	doi:10.5061/dryad.326r8/67.	110.6	doi:10.5061/dryad.326r8/68.	316.0
*Trachyrincus murrayi*	SAMEA4028782	doi:10.5061/dryad.326r8/125.	132.6	doi:10.5061/dryad.326r8/126.	326.5
*Mora moro*	SAMEA4028783	doi:10.5061/dryad.326r8/43.	103.2	doi:10.5061/dryad.326r8/44.	292.2
*Laemonema laureysi*	SAMEA4028784	doi:10.5061/dryad.326r8/45.	92.3	doi:10.5061/dryad.326r8/46.	266.4
*Bathygadus melanobranchus*	SAMEA4028785	doi:10.5061/dryad.326r8/37.	129.8	doi:10.5061/dryad.326r8/38.	285.2
*Macrourus berglax*	SAMEA4028786	doi:10.5061/dryad.326r8/33.	120.9	doi:10.5061/dryad.326r8/34.	312.6
*Malacocephalus occidentalis*	SAMEA4028787	doi:10.5061/dryad.326r8/35.	106.0	doi:10.5061/dryad.326r8/36.	230.5
*Phycis blennoides*	SAMEA4028788	doi:10.5061/dryad.326r8/127.	121.7	doi:10.5061/dryad.326r8/128.	455.0
*Phycis phycis*	SAMEA4028789	doi:10.5061/dryad.326r8/17.	104.6	doi:10.5061/dryad.326r8/18.	240.8
*Lota lota*	SAMEA4028790	doi:10.5061/dryad.326r8/21.	119.9	doi:10.5061/dryad.326r8/22.	254.5
*Molva molva*	SAMEA4028791	doi:10.5061/dryad.326r8/19.	131.6	doi:10.5061/dryad.326r8/20.	243.0
*Brosme brosme*	SAMEA4028792	doi:10.5061/dryad.326r8/23.	125.0	doi:10.5061/dryad.326r8/24.	251.4
*Trisopterus minutus*	SAMEA4028793	doi:10.5061/dryad.326r8/5.	101.5	doi:10.5061/dryad.326r8/6.	269.0
*Gadiculus argenteus*	SAMEA4028794	doi:10.5061/dryad.326r8/15.	119.2	doi:10.5061/dryad.326r8/16.	291.8
*Pollachius virens*	SAMEA4028795	doi:10.5061/dryad.326r8/7.	120.2	doi:10.5061/dryad.326r8/8.	230.0
*Melanogrammus aeglefinus*	SAMEA4028796	doi:10.5061/dryad.326r8/9.	114.6	doi:10.5061/dryad.326r8/10.	256.7
*Merlangius merlangus*	SAMEA4028797	doi:10.5061/dryad.326r8/11.	128.6	doi:10.5061/dryad.326r8/12.	284.2
*Arctogadus glacialis*	SAMEA4028798	doi:10.5061/dryad.326r8/1.	130.1	doi:10.5061/dryad.326r8/2.	286.9
*Boreogadus saida*	SAMEA4028799	doi:10.5061/dryad.326r8/3.	124.9	doi:10.5061/dryad.326r8/4.	290.5
*Theragra chalcogramma*	SAMEA4028800	doi:10.5061/dryad.326r8/13.	135.8	doi:10.5061/dryad.326r8/14.	304.1
*Gadus morhua*	SAMEA4028801	doi:10.5061/dryad.326r8/131.	146.9	doi:10.5061/dryad.326r8/132.	336.6
*Regalecus glesne*	SAMEA4028802	doi:10.5061/dryad.326r8/73.	200.3	doi:10.5061/dryad.326r8/74.	312.1
*Lampris guttatus*	SAMEA4028803	doi:10.5061/dryad.326r8/75.	259.5	doi:10.5061/dryad.326r8/76.	671.2
*Monocentris japonica*	SAMEA4028804	doi:10.5061/dryad.326r8/99.	169.7	doi:10.5061/dryad.326r8/100.	283.4
*Myripristis jacobus*	SAMEA4028805	doi:10.5061/dryad.326r8/63.	220.3	doi:10.5061/dryad.326r8/64.	319.3
*Holocentrus rufus*	SAMEA4028806	doi:10.5061/dryad.326r8/65.	198.9	doi:10.5061/dryad.326r8/66.	307.2
*Neoniphon sammara*	SAMEA4028807	doi:10.5061/dryad.326r8/97.	201.5	doi:10.5061/dryad.326r8/98.	278.7
*Beryx splendens*	SAMEA4028808	doi:10.5061/dryad.326r8/95.	163.5	doi:10.5061/dryad.326r8/96.	572.3
*Rondeletia loricata*	SAMEA4028809	doi:10.5061/dryad.326r8/93.	173.6	doi:10.5061/dryad.326r8/94.	450.5
*Acanthochaenus luetkenii*	SAMEA4028810	doi:10.5061/dryad.326r8/101.	167.4	doi:10.5061/dryad.326r8/102.	350.9
*Brotula barbata*	SAMEA4028811	doi:10.5061/dryad.326r8/59.	148.2	doi:10.5061/dryad.326r8/60.	210.3
*Lamprogrammus exutus*	SAMEA4028812	doi:10.5061/dryad.326r8/57.	151.5	doi:10.5061/dryad.326r8/58.	482.8
*Carapus acus*	SAMEA4028813	doi:10.5061/dryad.326r8/61.	118.5	doi:10.5061/dryad.326r8/62.	200.3
*Chatrabus melanurus*	SAMEA4028814	doi:10.5061/dryad.326r8/69.	347.3	doi:10.5061/dryad.326r8/70.	1001.0
*Thunnus albacares*	SAMEA4028815	doi:10.5061/dryad.326r8/107.	222.2	doi:10.5061/dryad.326r8/108.	363.4
*Lesueurigobius cf. sanzoi*	SAMEA4028816	doi:10.5061/dryad.326r8/129.	244.7	doi:10.5061/dryad.326r8/120.	683.5
*Perca fluviatilis*	SAMEA4028817	doi:10.5061/dryad.326r8/83.	193.8	doi:10.5061/dryad.326r8/84.	382.4
*Myoxocephalus scorpius*	SAMEA4028818	doi:10.5061/dryad.326r8/123.	158.7	doi:10.5061/dryad.326r8/124.	375.4
*Sebastes norvegicus*	SAMEA4028819	doi:10.5061/dryad.326r8/85.	219.4	doi:10.5061/dryad.326r8/86.	339.5
*Chaenocephalus aceratus*	SAMEA4028820	doi:10.5061/dryad.326r8/87.	190.5	doi:10.5061/dryad.326r8/88.	573.8
*Symphodus melops*	SAMEA4028821	doi:10.5061/dryad.326r8/119.	162.9	doi:10.5061/dryad.326r8/120.	238.1
*Spondyliosoma cantharus*	SAMEA4028822	doi:10.5061/dryad.326r8/105.	209.0	doi:10.5061/dryad.326r8/106.	281.5
*Antennarius striatus*	SAMEA4028823	doi:10.5061/dryad.326r8/79.	135.7	doi:10.5061/dryad.326r8/80.	280.1
*Selene dorsalis*	SAMEA4028824	doi:10.5061/dryad.326r8/113.	161.7	doi:10.5061/dryad.326r8/114.	235.8
*Helostoma temminckii*	SAMEA4028825	doi:10.5061/dryad.326r8/109.	183.5	doi:10.5061/dryad.326r8/110.	241.8
*Anabas testudineus*	SAMEA4028826	doi:10.5061/dryad.326r8/111.	160.6	doi:10.5061/dryad.326r8/112.	206.3
*Parablennius parvicornis*	SAMEA4028827	doi:10.5061/dryad.326r8/117.	182.7	doi:10.5061/dryad.326r8/118.	257.3
*Chromis chromis*	SAMEA4028828	doi:10.5061/dryad.326r8/115.	253.3	doi:10.5061/dryad.326r8/116.	397.6
*Pseudochromis fuscus*	SAMEA4028829	doi:10.5061/dryad.326r8/121.	199.4	doi:10.5061/dryad.326r8/122.	284.4

**Table 5:** Gene space completeness metrics for all draft assemblies in this data set (Data Citation 2)

**Species**	CEGMA complete*	CEGMA partial*	**BUSCO complete**†	**BUSCO duplicated**†	**BUSCO fragmented**†	**BUSCO missing**†
*Osmerus eperlanus*	175	220	2,071	71	760	867
*Borostomias antarcticus*	116	180	1,101	37	869	1,728
*Parasudis fraserbrunneri*	131	205	1,625	63	880	1,193
*Guentherus altivela*	44	119	402	11	809	2,487
*Benthosema glaciale*	171	214	1,478	128	768	1,452
*Polymixia japonica*	188	230	2,474	80	663	561
*Percopsis transmontana*	185	233	2,411	71	639	648
*Typhlichthys subterraneus*	167	222	2,024	55	763	911
*Zeus faber*	155	219	1,758	48	917	1,023
*Cyttopsis roseus*	185	233	1,969	62	867	862
*Stylephorus chordatus*	125	193	1,189	39	926	1,583
*Bregmaceros cantori*	85	189	867	32	942	1,889
*Merluccius polli*	133	204	1,188	44	1,019	1,491
*Merluccius merluccius*	147	210	1,257	36	959	1,482
*Merluccius capensis*	144	209	1,363	38	986	1,349
*Melanonus zugmayeri*	163	218	1,803	56	895	1,000
*Muraenolepis marmoratus*	143	209	1,258	37	1,040	1,400
*Trachyrincus scabrus*	190	225	1,957	45	872	869
*Trachyrincus murrayi*	218	235	2,806	69	464	428
*Mora moro*	155	215	1,582	40	948	1,168
*Laemonema laureysi*	163	223	1,814	41	844	1,040
*Bathygadus melanobranchus*	179	223	2,026	60	831	841
*Macrourus berglax*	147	206	1,172	61	927	1,599
*Malacocephalus occidentalis*	147	210	1,419	40	996	1,283
*Phycis blennoides*	190	231	2,155	44	717	826
*Phycis phycis*	144	213	1,461	36	966	1,271
*Lota lota*	169	213	1,740	49	902	1,056
*Molva molva*	172	218	1,739	61	939	1,020
*Brosme brosme*	167	215	1,712	36	907	1,079
*Trisopterus minutus*	138	189	1,199	39	917	1,582
*Gadiculus argenteus*	119	195	1,193	26	958	1,547
*Pollachius virens*	147	208	1,405	35	968	1,325
*Melanogrammus aeglefinus*	145	201	1,356	41	996	1,346
*Merlangius merlangus*	136	195	1,429	39	946	1,323
*Arctogadus glacialis*	141	199	1,177	34	1,036	1,485
*Boreogadus saida*	137	205	1,261	43	955	1,482
*Theragra chalcogramma*	153	210	1,486	36	971	1,241
*Gadus morhua*	202	235	2,455	43	608	635
*Regalecus glesne*	184	234	2,228	70	675	795
*Lampris guttatus*	129	201	1,467	47	953	1,278
*Monocentris japonica*	199	235	2,709	74	545	444
*Myripristis jacobus*	200	230	2,844	102	461	393
*Holocentrus rufus*	202	233	2,944	97	441	313
*Neoniphon sammara*	195	228	2,742	81	505	451
*Beryx splendens*	152	197	1,638	58	941	1,119
*Rondeletia loricata*	140	204	1,828	62	921	949
*Acanthochaenus luetkenii*	138	219	1,736	59	936	1,026
*Brotula barbata*	230	243	3,278	97	247	173
*Lamprogrammus exutus*	146	211	1,692	77	1,011	995
*Carapus acus*	215	240	2,666	52	505	527
*Chatrabus melanurus*	92	188	1,142	36	1,091	1,465
*Thunnus albacares*	209	236	3,147	99	351	200
*Lesueurigobius* cf.* sanzi*	163	206	2,130	58	625	943
*Perca fluviatilis*	122	189	1,673	55	1,027	998
*Myoxocephalus scorpius*	164	217	2,016	69	803	879
*Sebastes norvegicus*	190	233	2,458	69	698	542
*Chaenocephalus aceratus*	146	199	1,918	63	876	904
*Symphodus melops*	199	229	2,755	76	537	406
*Spondyliosoma cantharus*	215	240	3,001	78	426	271
*Antennarius striatus*	195	226	2,312	73	656	730
*Selene dorsalis*	215	231	2,968	80	427	303
*Helostoma temminckii*	225	236	3,387	100	204	107
*Anabas testudineus*	225	240	3,314	110	245	139
*Parablennius parvicornis*	186	224	2,336	63	650	712
*Chromis chromis*	180	230	2,451	75	670	577
*Pseudochromis fuscus*	210	231	2,837	90	475	386

*Out of 248 highly conserved eukaryotic genes.

^†^Out of 3,698 highly conserved acanthopterygian genes.

**Table 6:** GenBank accession numbers for 120 previously published mitochondrial genomes (Data Citation 5 – 124)

**Species**	**GenBank accession**	**Species**	**GenBank accession**
*Abudefduf vaigiensis*	NC_009064	*Kareius bicoloratus*	NC_003176
*Allocyttus niger*	NC_004398	*Labracinus cyclophthalmus*	NC_009054
*Anabas testudineus*	NC_024752	*Lampris guttatus*	NC_003165
*Anomalops katoptron*	NC_008128	*Lamprogrammus niger*	NC_004378
*Anoplogaster cornuta*	NC_004391	*Lophiomus setigerus*	NC_008125
*Antennarius striatus*	AB282828	*Lophius americanus*	NC_004380
*Antigonia capros*	NC_004391	*Lota lota*	NC_004379
*Aphredoderus sayanus*	NC_004372	*Lycodes toyamensis*	NC_004409
*Aptocyclus ventricosus*	NC_008129	*Mastacembelus favus*	NC_003193
*Arcos* sp KU 149	NC_004413	*Melanocetus murrayi*	NC_004384
*Aspasma minima*	NC_008130	*Melanotaenia lacustris*	NC_004385
*Ateleopus japonicus*	NC_003178	*Monocentris japonicus*	NC_004392
*Aulopus japonicus*	NC_002674	*Monopterus albus*	NC_003192
*Bassozetus zenkevitchi*	NC_004374	*Mugil cephalus*	NC_003182
*Batrachomoeus trispinosus*	AP006738	*Myctophum affine*	NC_003163
*Beryx decadactylus*	NC_004393	*Myripristis berndti*	NC_003189
*Beryx splendens*	NC_003188	*Neocyttus rhomboidalis*	NC_004399
*Bregmaceros nectabanus*	NC_008124	*Neolamprologus brichardi*	NC_009062
*Carangoides armatus*	NC_004405	*Neoscopelus microchir*	NC_003180
*Caranx melampygus*	NC_004406	*Odax cyanomelas*	NC_009061
*Carapus bermudensis*	NC_004373	*Oncorhynchus mykiss*	NC_001717
*Cataetyx rubrirostris*	NC_004375	*Oryzias latipes*	NC_004387
*Caulophryne jordani*	NC_004383	*Osmerus mordax*	NC_015246
*Cetostoma regani*	NC_004389	*Ostichthys japonicus*	NC_004394
*Champsocephalus gunnari*	NC_018340	*Paralichthys olivaceus*	NC_002386
*Chauliodus sloani*	NC_003159	*Parazen pacificus*	NC_004396
*Chaunax abei*	NC_004381	*Perca fluviatilis*	NC_026313
*Chaunax tosaensis*	NC_004382	*Percopsis transmontana*	NC_003168
*Chlorophthalmus agassizi*	NC_003160	*Petroscirtes breviceps*	NC_004411
*Coelorinchus kishinouyei*	NC_003169	*Pholis crassispina*	NC_004410
*Cololabis saira*	NC_003183	*Physiculus japonicus*	NC_004377
*Coregonus lavaretus*	NC_002646	*Polymixia japonica*	NC_002648
*Cottus reinii*	NC_004404	*Polymixia lowei*	NC_003181
*Crossostoma lacustre*	NC_001727	*Porichthys myriaster*	NC_006920
*Cyprinus carpio*	NC_001606	*Poromitra oscitans*	NC_003172
*Dactyloptena peterseni*	NC_003194	*Pterocaesio tile*	NC_004408
*Dactyloptena tiltoni*	NC_004402	*Rhyacichthys aspro*	NC_004414
*Danacetichthys galathenus*	NC_003185	*Rondeletia loricata*	NC_003186
*Diaphus splendidus*	NC_003164	*Salarias fasciatus*	AP004451
*Diplacanthopoma brachysoma*	NC_004376	*Sardinops melanostictus*	NC_002616
*Diplophos* sp MM1999	AB034825	*Sargocentron rubrum*	NC_004395
*Diretmoides veriginae*	NC_008126	*Satyrichthys amiscus*	NC_004403
*Diretmus argenteus*	NC_008127	*Saurida undosquamis*	NC_003162
*Eleotris acanthopoma*	NC_004415	*Scarus schlegeli*	NC_011936
*Emmelichthys struhsakeri*	NC_004407	*Scomber japonicus*	NC_013723
*Etheostoma radiosum*	NC_005254	*Scopelogadus mizolepis*	NC_003171
*Exocoetus volitans*	NC_003184	*Sigmops gracilis*	NC_002574
*Gadus morhua*	NC_002081	*Sirembo imberbis*	NC_008123
*Gambusia affinis*	NC_004388	*Sparus aurata*	NC_024236
*Gasterosteus aculeatus*	AP002944	*Stephanolepis cirrhifer*	NC_003177
*Halieutaea stellata*	AP005977	*Stylephorus chordatus*	NC_009948
*Harpadon microchir*	NC_003161	*Sufflamen fraenatum*	NC_004416
*Helicolenus hilgendorfi*	NC_003195	*Synbranchus marmoratus*	AP004439
*Helostoma temminkii*	NC_022728	*Thunnus thynnus*	NC_014052
*Histrio histrio*	AB282829	*Trachipterus trachipterus*	NC_003166
*Hoplostethus japonicus*	NC_003187	*Zalieutes elater*	AB282835
*Hypoatherina tsurugae*	NC_004386	*Zenion japonicum*	NC_004397
*Hypoptychus dybowskii*	NC_004400	*Zenopsis nebulosus*	NC_003173
*Ijimaia dofleini*	NC_003179	*Zeus faber*	NC_003190
*Indostomus paradoxus*	NC_004401	*Zu cristatus*	NC_003167

**Table 7:** ID of all unitigs containing mitochondrial data for all species included in the data set

**Species**	**UTG IDs**
*Acanthochaenus luetkenii*	utg7180003914080, utg7180003914081
*Anabas testudineus*	utg7180000074085
*Antennarius striatus*	utg7180002097916, utg7180002097917, utg7180002097918, utg7180002097919
*Arctogadus glacialis*	utg7180001210258
*Bathygadus melanobranchus*	utg7180000000032
*Benthosema glaciale*	utg7180006223522, utg7180007030062, utg7180007030067, utg7180007030068, utg7180007152696, utg7180007434654, utg7180007609485, utg7180007660002, utg7180007660003, utg7180007673377, utg7180007673378
*Beryx splendens*	utg7180000469666, utg7180000771701, utg7180004939694, utg7180005165554, utg7180005165555, utg7180005341444, utg7180005341445, utg7180005366167, utg7180005385228, utg7180005385229, utg7180005385230, utg7180005385231, utg7180005476828, utg7180005476831, utg7180005476832, utg7180005569875, utg7180005569876, utg7180005673983, utg7180005673984, utg7180005673985, utg7180005691644, utg7180005691645, utg7180005705333, utg7180005869836, utg7180006066164, utg7180006120309, utg7180006133924
*Boreogadus saida*	utg7180001220567, utg7180001220611
*Borostomias antarcticus*	utg7180001274025, utg7180003691481, utg7180003691544, utg7180003691567, utg7180003754703, utg7180003754717, utg7180003754718, utg7180003754733, utg7180003811941, utg7180004025360, utg7180004025368, utg7180004025384, utg7180004102570, utg7180004469635, utg7180004469636, utg7180004492307, utg7180004584377, utg7180004605154, utg7180004605155, utg7180004625717
*Bregmaceros cantori*	utg7180000000000, utg7180000000028, utg7180000003257
*Brosme brosme*	utg7180001047115, utg7180001047140
*Brotula barbata*	utg7180000000018
*Carapus acus*	utg7180000000000
*Chaenocephalus aceratus*	utg7180002324592
*Chatrabus melanurus*	utg7180004205210, utg7180004208747, utg7180004208748, utg7180004208749, utg7180004208753, utg7180004208754, utg7180004208755, utg7180004208756, utg7180004212026, utg7180004212030, utg7180004212034, utg7180004212035, utg7180004212036, utg7180004215531, utg7180004215533,
*Chromis chromis*	utg7180001202954, utg7180001209662, utg7180001209663, utg7180001266383, utg7180001266389, utg7180001322771, utg7180001335484, utg7180001335485, utg7180001335486, utg7180001335489, utg7180001415702, utg7180001623875, utg7180001623880, utg7180001660412
*Cyttopsis roseus*	utg7180001278658, utg7180001278979
*Gadiculus argenteus*	utg7180001379789, utg7180001379798, utg7180001387987
*Guentherus altivela*	utg7180000271871, utg7180000494472, utg7180000503520, utg7180001132842, utg7180001368068, utg7180001512825, utg7180001890828, utg7180002011637, utg7180002196109, utg7180002773145, utg7180007337091, utg7180008012272, utg7180008012273, utg7180008012274, utg7180008012275, utg7180008692799, utg7180008692802, utg7180008940047, utg7180008940048, utg7180008940049, utg7180009466012
*Helostoma temminckii*	utg7180000715927, utg7180000715930
*Holocentrus rufus*	utg7180000000000
*Laemonema laureysi*	utg7180001371677
*Lampris guttatus*	utg7180002509326, utg7180002509328, utg7180002509329, utg7180002509330, utg7180002509333, utg7180002509335, utg7180002509336, utg7180002509337, utg7180002509339, utg7180002509340, utg7180002509341, utg7180002509342, utg7180002511349, utg7180002511350, utg7180002511351, utg7180002512148, utg7180002817224
*Lamprogrammus exutus*	utg7180004205210, utg7180004208747, utg7180004208748, utg7180004208749, utg7180004208753, utg7180004208754, utg7180004208755, utg7180004208756, utg7180004212026, utg7180004212030, utg7180004212034, utg7180004212035, utg7180004212036, utg7180004215531, utg7180004215533,
*Lesueurigobius cf. sanzoi*	utg7180000000879
*Lota lota*	utg7180000000000
*Macrourus berglax*	utg7180000034506, utg7180001621271, utg7180001623489, utg7180001624139
*Malacocephalus occidentalis*	utg7180000000000
*Melanogrammus aeglefinus*	utg7180000000000
*Melanonus zugmayeri*	utg7180000000010, utg7180001692953
*Merlangius merlangus*	utg7180000000000
*Merluccius capensis*	utg7180001513810
*Merluccius merluccius*	utg7180001887176, utg7180001917684, utg7180001972531, utg7180001981428, utg7180002025406, utg7180002025422, utg7180002097733, utg7180002097734, utg7180002097738, utg7180002079715, utg7180002146127, utg7180002218855, utg7180002218856, utg7180002307512
*Merluccius polli*	utg7180001442827
*Molva molva*	utg7180000000000
*Monocentris japonica*	utg7180000342919, utg7180000463143, utg7180000514479, utg7180000538369, utg7180001377029, utg7180001377031, utg7180001377032, utg7180001434412, utg7180001434417, utg7180001434418, utg7180001434429, utg7180001434430, utg7180001434446, utg7180001434447, utg7180001434448, utg7180001469573, utg7180001469581, utg7180001469582, utg7180001469586, utg7180001469587, utg7180001469589, utg7180001486715, utg7180001516364, utg7180001516372, utg7180001516373, utg7180001516374, utg7180001524523, utg7180001524552, utg7180001550906, utg7180001550907, utg7180001550908, utg7180001692212, utg7180001692214, utg7180001820920
*Mora moro*	utg7180000000000
*Muraenolepis marmoratus*	utg7180000000000, utg7180001973851, utg7180001973898
*Myoxocephalus scorpius*	utg7180002464675, utg7180002464676, utg7180002464677, utg7180002464678, utg7180002504481, utg7180002504482, utg7180002533598, utg7180002533599, utg7180002533605, utg7180002533606
*Myripristis jacobus*	utg7180000000000
*Neoniphon sammara*	utg7180000064300
*Osmerus eperlanus*	utg7180000000726
*Parablennius parvicornis*	utg7180000020269
*Parasudis fraserbrunneri*	utg7180003294189, utg7180003426264, utg7180003433528
*Perca fluviatilis*	utg7180001412776, utg7180001412933
*Percopsis transmontana*	utg7180000622724, utg7180000622787, utg7180000622789, utg7180000630769, utg7180000630770, utg7180000634249, utg7180000640180, utg7180000671918, utg7180000671919, utg7180000681531, utg7180000690201, utg7180000690202, utg7180000695306, utg7180000695308, utg7180000716419, utg7180000757684, utg7180000782025
*Phycis blennoides*	utg7180003799308
*Phycis phycis*	utg7180001189424
*Pollachius virens*	utg7180000000000
*Polymixia japonica*	utg7180001067565, utg7180001067570, utg7180001067565
*Pseudochromis fuscus*	utg7180001142570, utg7180001142583, utg7180001142600, utg7180001145451, utg7180001145455
*Regalecus glesne*	utg7180000000000
*Rondeletia loricata*	utg7180000491946, utg7180000516073, utg7180000842519, utg7180000847838, utg7180000928011, utg7180000966600, utg7180001048288, utg7180001149138, utg7180001161638, utg7180001206941, utg7180001478759, utg7180001623954, utg7180002730734, utg7180002730736, utg7180002730737, utg7180002814675, utg7180002976817, utg7180002976819, utg7180003297619, utg7180003297620, utg7180003394061, utg7180003394062, utg7180003438009, utg7180003438010, utg7180003438011, utg7180003438012, utg7180003438013, utg7180003936305, utg7180003936306, utg7180003936728, utg7180004045904, utg7180004045909, utg7180004317827, utg7180004338610, utg7180004338611, utg7180004601252, utg7180004621852, utg7180004746867, utg7180004746868
*Sebastes norvegicus*	utg7180001468849
*Selene dorsalis*	utg7180001234455
*Spondyliosoma cantharus*	utg7180001069401, utg7180001069405
*Stylephorus chordatus*	utg7180003402356, utg7180003402376, utg7180003402383, utg7180003402389, utg7180003428557, utg7180003428577, utg7180003428590, utg7180003428591, utg7180003428594, utg7180003428603, utg7180003428622, utg7180003444599, utg7180003444601, utg7180003444608, utg7180003456661, utg7180003456662, utg7180003456664, utg7180003456684, utg7180003456692, utg7180003514255, utg7180003514256, utg7180003560601, utg7180003560603, utg7180003560604, utg7180003560606, utg7180003560623, utg7180003727526, utg7180003727530, utg7180003727534, utg7180003727536, utg7180003727543, utg7180003727550, utg7180003727562, utg7180003917108, utg7180003917109, utg7180003917110, utg7180003917115, utg7180003917116, utg7180003917712, utg7180003917713, utg7180004070138, utg7180004070139
*Symphodus melops*	utg7180000868836, utg7180000889427, utg7180000868836
*Theragra chalcogramma*	utg7180000000000
*Thunnus albacares*	utg7180001817200, utg7180001817201, utg7180001817221, utg7180001817255, utg7180001817266, utg7180001817279, utg7180001817286, utg7180001817289, utg7180001817293, utg7180001958519, utg7180001958520, utg7180002005352, utg7180002005353, utg7180002005358, utg7180002030942, utg7180002030943, utg7180002031021, utg7180002031022, utg7180002163410, utg7180002163441
*Trachyrincus murrayi*	utg7180002283202, utg7180002334643
*Trachyrincus scabrus*	utg7180000000000
*Trisopterus minutus*	utg7180000000029
*Typhlichthys subterraneus*	utg7180000000573, utg7180000802674
*Zeus faber*	utg7180000003061, utg7180001638560

**Table 8:** Nuclear markers used in phylogenetic analyses

**ENSEMBL Gene ID**	**Gene name**	**Chr. in** ***D. rerio***	**Num. exons**	**Alignment length**	**Num. variable sites**	Num. PI sites*	**Proportion of missing data**	**Mean node support**	**SH** ***P*****-value**†	***K*****-score**
ENSDARG00000003189	psmd1	chr 22	6	730	116	75	0.025	0.62	0.013	439.5
ENSDARG00000003495	madd	chr 7	5	594	140	86	0.091	0.502	0.007	419.9
ENSDARG00000003984	LTN1	chr 10	4	472	183	120	0.115	0.518	0.006	352.7
ENSDARG00000004302	slc45a1	chr 11	4	524	122	82	0.047	0.55	0.025	425.8
ENSDARG00000005058	ncapd2	chr 2	7	820	357	221	0.079	0.683	0.008	360.2
ENSDARG00000005236	srcap	chr 12	8	910	261	177	0.059	0.791	0.002	361.5
ENSDARG00000006169	lrrk2	chr 25	3	330	140	83	0.105	0.502	0.006	450.5
ENSDARG00000007092	xab2	chr 3	4	466	135	84	0.042	0.52	0.015	410.9
ENSDARG00000007744	tsr1	chr 15	4	578	320	220	0.118	0.703	0.003	323.5
ENSDARG00000009953	med14	chr 9	4	444	126	72	0.096	0.628	0.001	NA
ENSDARG00000009965	mag	chr 15	4	792	309	207	0.038	0.744	0.002	286.9
ENSDARG00000011764	asun	chr 4	3	376	121	80	0.104	0.492	0.013	434.1
ENSDARG00000012403	ERCC6L2	chr 8	4	456	239	176	0.081	0.684	0.002	296.2
ENSDARG00000013150	dhx16	chr 15	5	606	184	123	0.111	0.611	0.002	387.9
ENSDARG00000013240	zgc172271	chr 6	5	534	282	193	0.082	0.567	0.003	308.8
ENSDARG00000016177	eif4enif1	chr 6	4	488	194	140	0.06	0.658	0.006	363.3
ENSDARG00000016415	dhtkd1	chr 25	4	516	237	161	0.084	0.658	0.013	311.4
ENSDARG00000016443	eif3c	chr 12	6	724	133	92	0.095	0.523	0.012	395.8
ENSDARG00000016775	aqr	chr 17	5	624	156	101	0.083	0.646	0.001	411.4
ENSDARG00000016936	hmcn1	chr 20	5	574	304	197	0.085	0.797	0.003	321.4
ENSDARG00000017034	sqrdl	chr 25	5	602	245	157	0.014	0.676	0.001	268
ENSDARG00000017696	diexf	chr 13	5	688	349	247	0.081	0.772	0.002	NA
ENSDARG00000018296	rev1	chr 9	3	350	173	110	0.065	0.562	0.037	NA
ENSDARG00000019000	smc3	chr 22	6	748	154	87	0.073	0.625	0.002	461.2
ENSDARG00000019300	ints7	chr 20	3	394	130	82	0.058	0.512	0.006	408.6
ENSDARG00000019834	EDRF1	chr 17	8	968	394	249	0.068	0.815	0.02	307.3
ENSDARG00000022730	aasdh	chr 20	4	462	251	186	0.076	0.607	0.016	289.8
ENSDARG00000025011	synj1	chr 10	8	998	243	172	0.076	0.79	0.001	338.7
ENSDARG00000025269	pdcd6ip	chr 19	3	372	171	121	0.119	0.437	0.006	NA
ENSDARG00000026180	prpf8	chr 15	14	1,874	173	128	0.018	0.791	0.007	388
ENSDARG00000027353	zmym2	chr 9	3	366	131	99	0.096	0.498	0.003	396.9
ENSDARG00000027689	pold1	chr 3	3	348	97	68	0.069	0.558	0.005	452.6
**ENSDARG00000029556**	**kansl3**	**chr 8**	**4**	**444**	**164**	**110**	**0.034**	**0.67**	**0.069**	**330.4**
ENSDARG00000030945	si:ch211-259g3.4	chr 15	11	1,888	777	490	0.104	0.874	0.003	317.6
ENSDARG00000031886	ift140	chr 24	4	474	265	176	0.097	0.659	0.007	363.3
ENSDARG00000032459	med24	chr 12	3	332	126	77	0.072	0.5	0.002	394.2
ENSDARG00000032704	qrsl1	chr 17	4	470	228	154	0.034	0.723	0.008	347.6
ENSDARG00000034178	cpsf1	chr 19	4	458	119	72	0.09	0.479	0.003	460.6
ENSDARG00000035330	taf1	chr 5	5	588	162	114	0.097	0.634	0.01	398.9
ENSDARG00000035761	mcm7	chr 14	4	426	140	98	0.059	0.609	0.005	397.7
ENSDARG00000035978	ube3c	chr 7	4	526	154	83	0.095	0.574	0.001	418.3
ENSDARG00000036338	vps11	chr 10	10	1,238	345	216	0.054	0.719	0.002	269.3
ENSDARG00000036755	prmt10	chr 1	5	604	248	158	0.038	0.65	0.003	344.6
ENSDARG00000037017	ube4b	chr 23	4	532	98	60	0.073	0.534	0.003	476.2
ENSDARG00000037898	ppl	chr 3	4	438	255	181	0.08	0.579	0.003	310.8
ENSDARG00000038882	smc4	chr 15	10	1,260	445	261	0.083	0.813	0.035	293.4
ENSDARG00000039134	MTR	chr 12	5	524	202	147	0.091	0.703	0.006	384.2
ENSDARG00000041895	cad	chr 20	6	790	200	124	0.088	0.582	0.005	355.1
ENSDARG00000042530	nup205	chr 18	13	1,536	599	377	0.051	0.844	0.028	246.1
ENSDARG00000042728	plaa	chr 7	5	582	249	164	0.074	0.609	0.014	NA
ENSDARG00000043019	exoc1	chr 20	4	528	137	83	0.017	0.606	0	351.6
ENSDARG00000045626	nek8	chr 15	3	428	78	54	0.032	0.49	0.002	478.6
ENSDARG00000045900	agbl5	chr 4	3	300	131	83	0.058	0.592	0.014	413.5
ENSDARG00000051889	dhodh	chr 7	5	566	233	171	0.072	0.669	0.012	287.7
ENSDARG00000053087	mthfr	chr 8	3	360	121	79	0.071	0.484	0.001	NA
ENSDARG00000053200	dis3l	chr 7	3	316	174	116	0.101	0.672	0.01	390.5
ENSDARG00000053303	map3k4	chr 13	4	458	170	100	0.084	0.481	0.003	388.6
ENSDARG00000054154	bms1l	chr 12	6	700	285	199	0.057	0.663	0.001	NA
**ENSDARG00000056037**	**itih6**	**chr 23**	**3**	**372**	**183**	**113**	**0.066**	**0.554**	**0.21**	**397.1**
ENSDARG00000056160	hspd1	chr 9	3	388	121	85	0.073	0.543	0	391.5
ENSDARG00000056318	kansl2	chr 23	4	428	172	112	0.056	0.65	0.004	346.3
ENSDARG00000056530	CPAMD8	chr 22	6	820	293	176	0.089	0.577	0.028	318.7
ENSDARG00000056932	tfip11	chr 23	3	390	131	91	0.097	0.448	0.006	NA
ENSDARG00000057508	nbeal2	chr 16	3	482	276	193	0.107	0.54	0.006	345.1
ENSDARG00000057997	tmf1	chr 11	3	308	151	105	0.053	0.583	0.009	NA
ENSDARG00000058533	pole	chr 5	7	848	234	147	0.076	0.663	0.018	NA
ENSDARG00000059553	SYMPK	chr 5	7	900	308	196	0.075	0.626	0.01	355.6
ENSDARG00000059631	ints1	chr 3	11	1,360	405	288	0.076	0.825	0.004	270.1
ENSDARG00000059711	nol6	chr 5	6	624	330	226	0.076	0.665	0.009	NA
ENSDARG00000059760	wdtc1	chr 16	3	386	132	85	0.025	0.662	0.004	354
ENSDARG00000059846	EPG5	chr 5	7	930	515	354	0.086	0.728	0.009	310.1
ENSDARG00000059925	usp24	chr 20	5	590	182	112	0.071	0.707	0	413.7
ENSDARG00000060089	btaf1	chr 13	7	764	300	196	0.078	0.781	0.022	235.2
ENSDARG00000061013	ankfy1	chr 5	6	726	203	127	0.068	0.653	0.013	340.2
ENSDARG00000061394	baz1b	chr 18	3	1,034	509	329	0.113	0.656	0.009	339.5
**ENSDARG00000061789**	**gnl1**	**chr 16**	**3**	**322**	**162**	**109**	**0.048**	**0.577**	**0.091**	**356.2**
ENSDARG00000062198	pcm1	chr 1	6	790	328	185	0.076	0.754	0.005	378.6
ENSDARG00000062632	duox	chr 25	11	1270	602	387	0.053	0.867	0.001	217.5
ENSDARG00000062868	eea1	chr 18	3	362	142	94	0.071	0.576	0.008	413.8
ENSDARG00000063558	ARHGEF17	chr 18	3	386	144	87	0.089	0.525	0.006	381.5
ENSDARG00000063626	ddx21	chr 13	4	424	177	120	0.033	0.713	0.001	359.9
ENSDARG00000067805	ggcx	chr 10	5	516	173	119	0.059	0.619	0	343
**ENSDARG00000069274**	**ighmbp2**	**chr 18**	**8**	**1,108**	**561**	**398**	**0.039**	**0.783**	**0.05**	**268.8**
ENSDARG00000070109	ncapg	chr 1	3	326	135	94	0.092	0.523	0.003	NA
ENSDARG00000071294	TONSL	chr 17	3	332	184	137	0.068	0.607	0.021	321.2
ENSDARG00000073862	ptpn13	chr 21	5	572	227	141	0.109	0.64	0.018	353.5
ENSDARG00000074137	C2CD3	chr 15	5	620	340	233	0.094	0.638	0.012	NA
ENSDARG00000074314	TTC37	chr 5	3	398	209	148	0.1	0.482	0.017	NA
ENSDARG00000074410	brip1	chr 15	3	334	146	106	0.097	0.571	0.002	NA
ENSDARG00000074424	ibtk	chr 16	4	548	249	163	0.097	0.564	0.017	327.2
ENSDARG00000074524	CNTNAP1	chr 3	9	1,186	439	309	0.039	0.862	0.001	292.3
ENSDARG00000074571	GPAA1	chr 2	5	586	183	120	0.105	0.515	0.011	302.9
ENSDARG00000074675	pan2	chr 23	5	758	230	137	0.064	0.761	0.002	382.5
ENSDARG00000074749	abca12	chr 9	4	560	274	192	0.096	0.737	0.009	270.1
ENSDARG00000074759	ccar1	chr 13	5	600	150	90	0.091	0.659	0.002	NA
ENSDARG00000075108	tmco3	chr 1	4	460	194	113	0.071	0.64	0.002	444.1
ENSDARG00000075672	pms2	chr 12	4	450	186	117	0.046	0.711	0.001	NA
ENSDARG00000075798	USP38	chr 1	3	674	332	208	0.05	0.572	0.047	NA
ENSDARG00000075826	msh4	chr 17	5	550	221	122	0.016	0.731	0.009	356.3
ENSDARG00000076920	ZNF335	chr 8	5	538	191	121	0.083	0.556	0.002	NA
ENSDARG00000076994	gpr124	chr 8	5	844	454	279	0.098	0.78	0.012	313.6
ENSDARG00000077139	col6a3	chr 9	3	994	577	407	0.115	0.668	0.003	318.3
ENSDARG00000077469	polr1b	chr 13	6	708	331	223	0.09	0.786	0.001	336.1
ENSDARG00000077536	snrnp200	chr 8	10	1,134	223	140	0.052	0.773	0.004	398.3
ENSDARG00000077860	ankhd1	chr 21	3	398	47	33	0.039	0.43	0.007	NA
ENSDARG00000077891	npc1l1	chr 2	4	486	206	148	0.097	0.729	0	384.1
ENSDARG00000078135	MRC2	chr 3	3	318	177	110	0.086	0.681	0.006	419.7
ENSDARG00000078890	wdfy3	chr 5	13	1,528	490	291	0.097	0.706	0.005	365.9
ENSDARG00000079702	wdr81	chr 15	3	342	132	75	0.066	0.545	0.002	370.6
ENSDARG00000079751	megf8	chr 16	12	1,498	571	336	0.097	0.703	0.028	291.5
ENSDARG00000090858	AREL1	chr 17	5	654	190	112	0.028	0.627	0.037	375.9

*The number of parsimony-informative sites.

^†^The *P*-values obtained from the Shimodaira-Hasegawa tests. Genes with non-significant *P*-value from the Shimodaira-Hasegawa tests are in bold.
